# Epidemiology and aetiology of maternal bacterial and viral infections in low- and middle-income countries

**Published:** 2011-12

**Authors:** Prasad Palani Velu, Courtney A. Gravett, Tom K. Roberts, Thor A. Wagner, Jian Shayne F. Zhang, Craig E. Rubens, Michael G. Gravett, Harry Campbell, Igor Rudan

**Affiliations:** 1 Centre for Population Health Sciences and Global Health Academy, The University of Edinburgh, Scotland, UK; 2 Global Alliance to Prevent Prematurity and Stillbirth (GAPPS), Seattle Children’s Hospital, Seattle, Washington, USA; 3 Department of Pediatrics, University of Washington, Seattle, Washington, USA; 4 Department of Obstetrics and Gynecology, University of Washington, Seattle, Washington, USA; * Joint first or senior authorship

## Abstract

**Background:**

Maternal morbidity and mortality in low- and middle-income countries has remained exceedingly high. However, information on bacterial and viral maternal infections, which are important contributors to poor pregnancy outcomes, is sparse and poorly characterised. This review aims to describe the epidemiology and aetiology of bacterial and viral maternal infections in low- and middle-income countries.

**Methods:**

A systematic search of published literature was conducted and data on aetiology and epidemiology of maternal infections was extracted from relevant studies for analysis. Searches were conducted in parallel by two reviewers (using OVID) in the following databases: Medline (1950 to 2010), EMBASE (1980 to 2010) and Global Health (1973 to 2010).

**Results:**

Data from 158 relevant studies was used to characterise the epidemiology of the 10 most extensively reported maternal infections with the following median prevalence rates: *Treponema pallidum* (2.6%), *Neisseria gonorrhoeae* (1.5%), *Chlamydia trachomatis* (5.8%), Group B *Streptococcus* (8.6%), bacterial vaginosis (20.9%), hepatitis B virus (4.3%), hepatitis C virus (1.4%), *Cytomegalovirus* (95.7% past infection), *Rubella* (8.9% susceptible) and *Herpes simplex* (20.7%). Large variations in the prevalence of these infections between countries and regions were noted.

**Conclusion:**

This review confirms the suspected high prevalence of maternal bacterial and viral infections and identifies particular diseases and regions requiring urgent attention in public health policy planning, setting research priorities and donor funding towards reducing maternal morbidity and mortality in low- and middle-income countries.

Maternal morbidity and mortality in low- and middle-income countries are still unacceptably high. It was estimated that 529 000 maternal deaths occurred throughout the world annually in 2000 (1). This estimate was recently updated with a figure of 273 500 deaths in 2011, the majority of which occurred in poor countries (2). The problem of maternal health has gained the attention of the global community, as exemplified by United Nations Millennium Development Goal (MDG) 5, which is aimed at reducing the maternal mortality ratio by three quarters and ensuring universal access to reproductive health care by 2015 (3). With only 5 years left to achieve MDGs, progress towards the maternal health MDG has been one of the most disappointing, leading to its being highlighted as an urgent global priority at the September 2010 UN Summit on MDGs (4).

The disparity in maternal health between the developed and developing world can be attributed largely to poor access and quality of reproductive health care in developing countries (5). As a result, maternal mortality in developing countries remains high due to largely preventable causes such as haemorrhage, hypertensive disorders, abortion related complications and sepsis/infection (6).

An estimated 9.7% of maternal deaths in Africa are due to puerperal sepsis (6). Bacterial and viral infections during pregnancy contribute towards maternal morbidity and mortality and are associated with adverse pregnancy outcomes including spontaneous abortion, stillbirth, prematurity and low birth weight. Furthermore, some infections can be transmitted vertically to neonates, leading to subsequent neonatal morbidity and mortality (7). Most maternal infections can be diagnosed and treated during pregnancy, preventing morbidity and mortality of both mother and child. The reduction of maternal infections in the developing world is highly dependent on the effective use of limited health resources to diagnose and treat these infections.

The planning of effective public health measures is currently limited by the lack of information available on the precise epidemiology and aetiology of bacterial and viral maternal infections. Lack of information can also negatively impact donor interest and international commitment. This review aims to summarize published literature on the aetiology and epidemiology of bacterial and viral maternal infections in low- and middle-income countries. Additionally, the review aims to identify gaps in available information on the subject. This epidemiological information can subsequently be used to identify similarities and differences in the causes of maternal infection within and between geographic regions, and to guide local and international public health initiatives to reduce the prevalence and burden of these infections.

## METHODS

### Literature search terms

Initial searches were conducted to identify suitable keywords and MeSH headings to use in the final search (**Table 1**). The search strategy was prepared with input from a librarian. Searches were conducted in parallel by two reviewers (using OVID) in the following databases on 1 August 2010:

**Table 1:** Search terms used to identify published articles on the prevalence and etiology of maternal infections in the developing world

exp Infection/
AND
exp Pregnancy/ OR exp Pregnancy Complications, Infectious/
AND
exp Developing Countries OR africa/ or africa, northern/ or algeria/ or egypt/ or libya/ or morocco/ or tunisia/ or “africa south of the sahara”/ or africa, central/ or cameroon/ or central african republic/ or chad/ or congo/ or “democratic republic of the congo”/ or gabon/ or africa, eastern/ or burundi/ or djibouti/ or eritrea/ or ethiopia/ or kenya/ or rwanda/ or somalia/ or sudan/ or tanzania/ or uganda/ or africa, southern/ or angola/ or botswana/ or lesotho/ or malawi/ or mozambique/ or namibia/ or south africa/ or swaziland/ or zambia/ or zimbabwe/ or africa, western/ or benin/ or burkina faso/ or cape verde/ or cote d'ivoire/ or gambia/ or ghana/ or guinea/ or guinea-bissau/ or liberia/ or mali/ or mauritania/ or niger/ or nigeria/ or senegal/ or sierra leone/ or togo/ or caribbean region/ or west indies/ or “antigua and barbuda”/ or cuba/ or dominica/ or dominican republic/ or grenada/ or guadeloupe/ or haiti/ or jamaica/ or martinique/ or “saint kitts and nevis”/ or saint lucia/ or “saint vincent and the grenadines”/ or central america/ or belize/ or costa rica/ or el salvador/ or guatemala/ or honduras/ or nicaragua/ or panama/ or latin america/ or mexico/ or south america/ or argentina/ or bolivia/ or brazil/ or chile/ or colombia/ or ecuador/ or french guiana/ or guyana/ or paraguay/ or peru/ or suriname/ or uruguay/ or venezuela/ or asia/ or asia, central/ or kazakhstan/ or kyrgyzstan/ or tajikistan/ or turkmenistan/ or uzbekistan/ or asia, southeastern/ or borneo/ or brunei/ or cambodia/ or east timor/ or indonesia/ or laos/ or malaysia/ or mekong valley/ or myanmar/ or philippines/ or thailand/ or vietnam/ or asia, western/ or bangladesh/ or bhutan/ or india/ or sikkim/ or middle east/ or afghanistan/ or iran/ or iraq/ or jordan/ or lebanon/ or syria/ or turkey/ or yemen/ or nepal/ or pakistan/ or sri lanka/ or far east/ or china/ or tibet/ or “democratic people's republic of korea”/ or mongolia/ or taiwan/ or atlantic islands/ or azores/ or albania/ or lithuania/ or bosnia-herzegovina/ or bulgaria/ or byelarus/ or “macedonia (republic)”/ or moldova/ or montenegro/ or romania/ or russia/ or bashkiria/ or dagestan/ or moscow/ or siberia/ or serbia/ or ukraine/ or yugoslavia/ or armenia/ or azerbaijan/ or “georgia (republic)”/ or indian ocean islands/ or comoros/ or madagascar/ or mauritius/ or reunion/ or seychelles/ or fiji/ or papua new guinea/ or vanuatu/ or guam/ or palau/ or “independent state of samoa”/ or tonga/

Medline (1950 to August Week 4 2010), EMBASE (1980 to 2010 Week 30) and Global Health (1973 to August 2010).

Study inclusion and exclusion criteria

Studies were screened by title and then by abstract for relevance. Studies were deemed relevant if they provided information on the aetiology or epidemiology of bacterial and viral infections in pregnant women in developing countries. These studies were then grouped according to pathogen studied, with some studies providing information on multiple pathogens. Studies providing information on the epidemiology of parasitic infections in pregnant women were identified but not analyzed, as they were addressed in a separate review. Studies reporting the prevalence of maternal HIV infection were identified but not included for analysis, as this information is available through other sources. Relevant English language papers were analyzed in this work, along with the Chinese electronic databases, with the intention of translating and analyzing non-English papers, too. The inclusion criteria were:

***Subjects****:* Pregnant women at any stage of pregnancy or labour, including the puerperium (up to 42 days after labour);

***Study location:*** Low- and middle-income countries (as defined by The World Bank in 2010);

***Study design and sampling methods:*** No restrictions applied;

***Data collection****:* Only studies that provided evidence of bacterial or viral infection using microbiological or serological test results were included;

***Results****:* Papers were selected if they provided information on the burden of a particular pathogen (the prevalence of a particular infection in pregnant women in the community over time/incidence) and/or the aetiology of bacterial and viral maternal infections (prevalence of a specific pathogen/infection).

### Quality criteria

Only studies with more than 500 subjects were included, because we wanted to protect strongly against implausible proportional contributions of certain pathogens which could have occurred due to chance in smaller data series. Papers were required to describe their samples and methods in detail, and provide microbiological or serological evidence of the aetiology of infection.

### Data extraction

Information on pathogen studied, sample population (pregnant women studied during pregnancy or at labour) and size, study setting, duration and type, microbiological/serological test used and results were extracted from abstracts and full papers for analysis.

### Data analysis

Epidemiology and aetiology of bacterial and viral maternal infections were summarized according to the pathogen studied. Only pathogens with 5 or more studies reporting on its epidemiology and/or aetiology were analyzed. Median prevalence of each infection was calculated and trends in the prevalence of maternal infections were noted.

### Selection of studies

**Figure 1:**
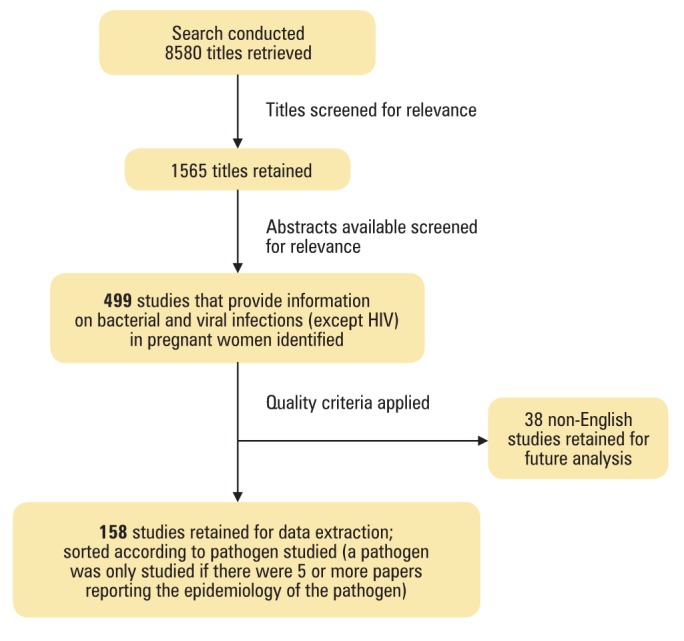
Summary of the literature search.

The final search yielded 8580 relevant titles. **Figure 1** outlines the results of the search process and application of inclusion and exclusion criteria, resulting in the final panel of studies from which data was extracted.

Studies retained for data extraction (n=158) characterized the prevalence of 5 bacterial pathogens (*Treponema pallidum, Neisseria gonorrhoeae, Chlamydia trachomatis,* Group B *Streptococcus*, bacterial vaginosis) and 5 viral pathogens (Hepatitis B virus, Hepatitis C virus, *Cytomegalovirus*, *Rubella*, *Herpes simplex*) among pregnant women in developing countries, with three further reports providing secondary cross-sectional insights or reviews of the literature in this field which were considered useful (8-168). Studies reporting prevalence maternal HIV infection (n=167) were not included in the analysis.

## RESULTS

### Prevalence of bacterial infections

**Figure 2:**
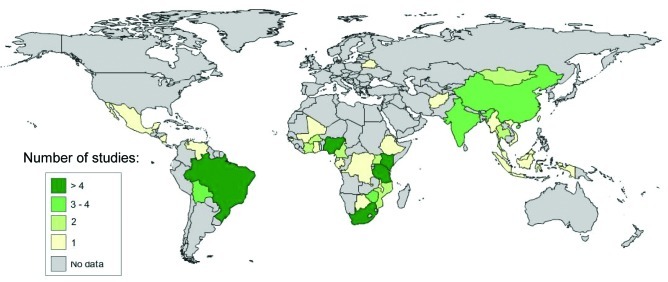
Geographical distribution of studies (n=72) reporting the prevalence of maternal syphilis; “no data” in the legend refers to low and middle-income countries only, as data from high-income countries were not the subject of this study.

**Figure 3:**
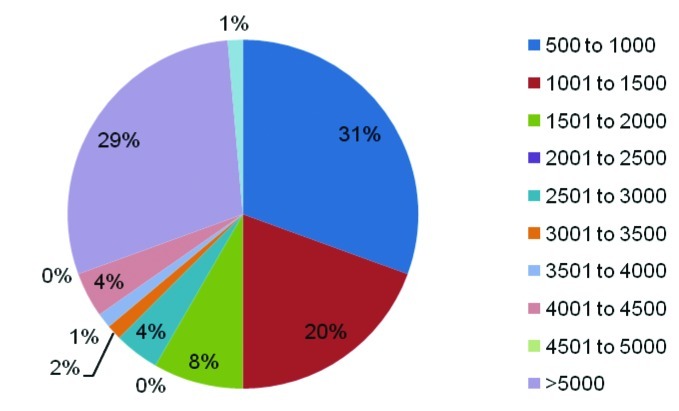
Distribution according to size of population studied in 72 studies reporting maternal syphilis prevalence.

**Figure 4:**
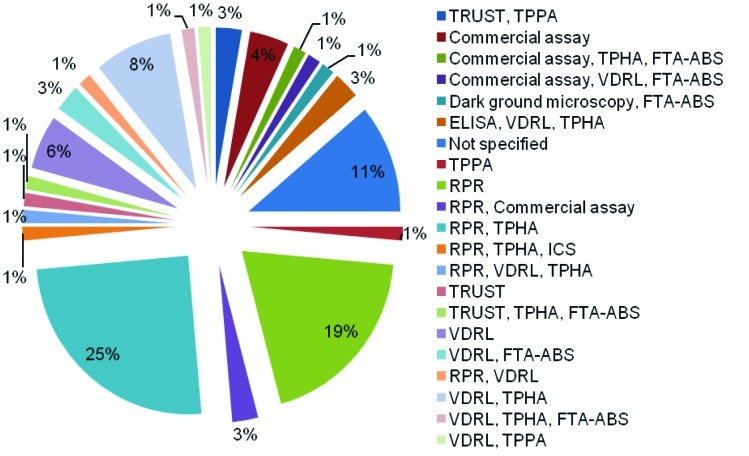
Techniques used to diagnose maternal syphilis in the 72 studies identified. (TRUST – Toluidine red unheated serum test; TPPA – *Treponema pallidum* particle agglutination test; TPHA – *Treponema pallidum* haemagglutination test; FTA-ABS – Fluorescent treponemal antibody absorption; VDRL – Venereal Disease Research Laboratory test; RPR – Rapid Plasma Reagent; ICS – immunochromatographic strip).

**Figure 5:**
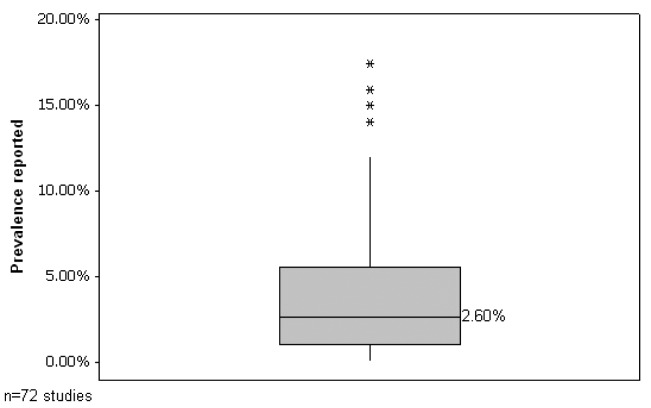
Box plot of syphilis prevalence reported by the 72 relevant studies. All studies measured prevalence by detecting the presence of antibodies towards *Treponema pallidum*. The following number summaries are depicted in the boxplot: the smallest observation (sample minimum), lower quartile (25%), median (50%), upper quartile (75%), and largest observation (sample maximum). Asterisks indicate outliers.

***Syphilis (Treponema pallidum).*** Seventy-two studies characterizing the prevalence of maternal syphilis in 36 developing countries were identified (**Supplementary Table 1**)(Supplementary Table 1). The features and results of these studies are summarised in **Figures 2**,******3**,******4******and******5**.

In terms of study design, 58.3% of the identified studies were cross-sectional, whilst 18.1% were screening studies. The majority of the studies (90.3%) were conducted in health care facilities (58.3% in antenatal or prenatal clinics), suggesting either awareness towards the need for antenatal screening for maternal syphilis infection, or merely that it is much easier to recruit study subjects in health care facilities. The remaining studies (5.6%) were community-based and the study setting was not specified in 4.2%.

Rapid Plasma Reagent (RPR) testing was used to detect anti-treponemal antibodies in many studies (49%), often in combination with another test, most commonly the *Treponema pallidum* Haemagglutination Assay (TPHA) (**Figure 4**). This is because RPR is cheap and simple to perform, but false-positive results are common, necessitating confirmatory testing (7). Particularly high prevalence of maternal syphilis was reported in studies from Cameroon, South Africa and Zimbabwe (around 15.0%). Both studies from Cameroon were conducted in the Yaounde province, and show an increase in the prevalence of maternal syphilis from 15.9% in 1992 to 17.4% in 1998 (17,66,107,130).

**Figure 6:**
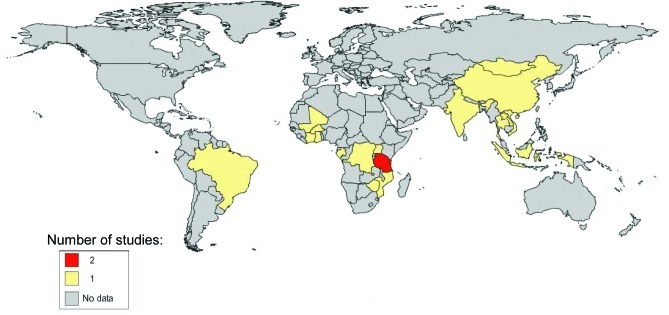
Geographical distribution of studies (n=21) reporting prevalence of maternal gonococcal infection; “no data” in the legend refers to low- and middle-income countries only, as data from high-income countries were not the subject of this study.

**Figure 7:**
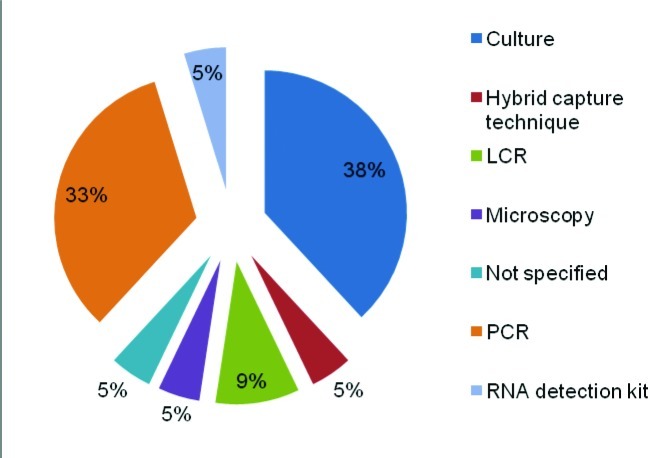
Techniques used to identify gonococcal infection in the 21 studies identified (LCR – ligase chain reaction).

**Figure 8:**
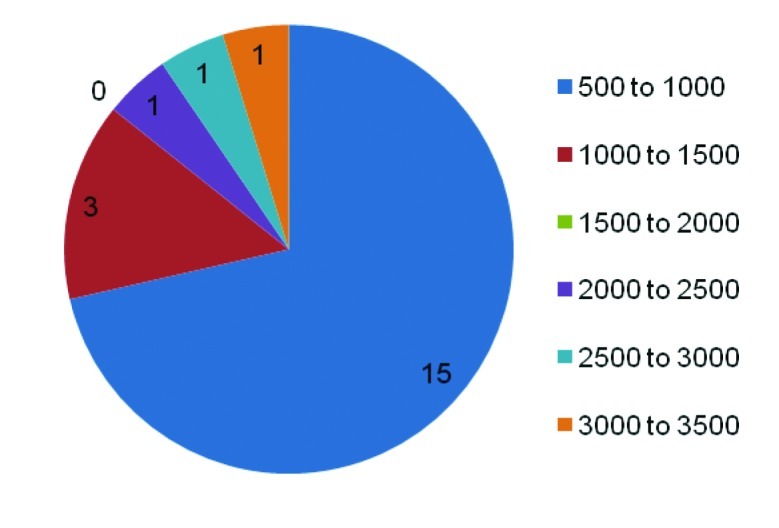
Size of study populations in 21 studies identified reporting maternal gonococcal infection prevalence.

**Figure 9:**
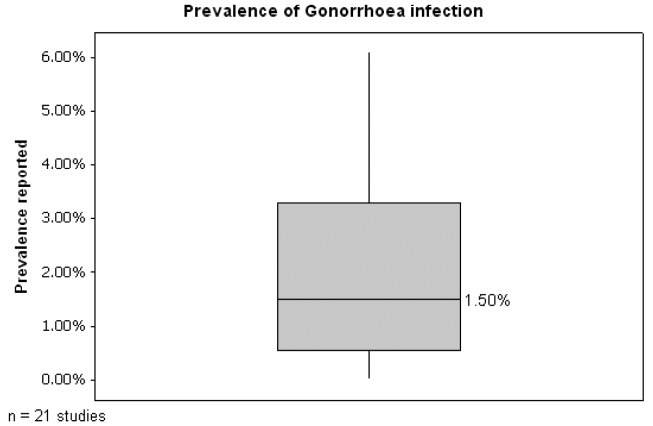
Prevalence of *N. gonorrhoeae* detected in relevant studies (n=21). All studies measured prevalence by detecting the infecting organism in pregnant women. The following number summaries are depicted in the boxplot: the smallest observation (sample minimum), lower quartile (25%), median (50%), upper quartile (75%), and largest observation (sample maximum).

***Gonorrhoea (Neisseria gonorrhoeae).*** Twenty-one studies providing information of the prevalence of maternal *Neisseria gonorrhoeae* infection were identified (**Supplementary Table 2**)(Supplementary Table 2). The characteristics of these studies and the prevalence reported are summarized in the **Figures 6**,******7**,******8******and******9**.

With regards to study design, 20 studies (95.2%) were cross-sectional and 1 was a randomized controlled trial (data from the control group was extracted). The majority of the studies (80.9%) were carried out in health care facilities and a minority was community-based (19.1%). Median prevalence of maternal gonococcal infection was relatively low, at 1.5% (**Figure 9**). However, higher prevalence of maternal gonococcal infection was reported in studies from Mongolia (6.1%), Vanuatu (5.9%) and Zimbabwe (5.8%) suggesting the need for targeted action in these countries (12,79,145).

***Chlamydia trachomatis.*** Nineteen studies reporting the prevalence of maternal *Chlamydia trachomatis* (CT) infection were identified (**Table 2**).

**Table 2:** Characteristics and results of studies (n=19) reporting prevalence of maternal *Chlamydia trachomatis* infection

Article	Location, setting of study	Type, duration of study	Population	Results / Prevalence	Technique used
Msuya *et al*, 2009 (100)	Tanzania, 2 primary health clinics	Cross-sectional study, 21 months	2654 pregnant women	17.5%	ELISA detecting anti-chlamydial IgG
Jalil *et al,* 2008 (63)	Brazil, prenatal services in 6 cities	Cross-sectional study, 1 year	3003 pregnant women	CT prevalence of 9.4%	Hybrid capture technique
Kinoshita-Moleka *et al*, 2008 (79)	Democratic Republic of Congo, 2 maternity clinics	Cross-sectional study, 4 months	529 pregnant women	1.7%	PCR
Lujan et al 2008 (89)	Mozambique, antenatal clinic	Cross-sectional study, 5 months	835 first void urine samples from pregnant women	4.1%	PCR
Romoren *et al*, 2007 (127)	Botswana, antenatal clinic	Cross-sectional study, singular time point	703 pregnant antenatal care attendees	8% prevalence	LCR
Chen *et al*, 2006 (27)	China, antenatal clinic	Cross-sectional study, 3 months	504 pregnant women	10.1%	PCR
Thammalangsy *et al*, 2006 (156)	Laos, 2 hospitals	Cross-sectional study, 7 months	500 antenatal attendees	10.2% by nucleic acid hybridisation and 9.6% by PCR	Nucleic acid hybridisation, PCR
Amindavaa *et al*, 2005 (2)	Mongolia, prenatal clinics	Cross-sectional survey, 11 months	2000 pregnant women	19.3%	PCR
Apea-Kubi *et al*, 2004 (14)	Ghana, gynaecology clinics at teaching hospital	Cross-sectional study, singular time point	517 pregnant women	3% prevalence	RNA detection kit
Sullivan *et al*, 2003 (145)	Vanuatu, antenatal clinic	Cross-sectional study, 12 months	547 pregnant women	21.5%	PCR
Gray *et al*, 2001 (53)	Uganda, community based	Randomised control trial, duration not specified	1576 pregnant women in the control arm of the study	2.7%	LCR
Mayank *et al*, 2001 (97)	India, community based	Cross-sectional study, duration not specified	600 pregnant women	4.3%	ELISA
Latif *et al*, 1999 (79)	Zimbabwe, Antenatal and primary care clinics	Cross-sectional study	1189 asymptomatic pregnant women	5.8% (and/or Gonococcal infection)	Not specified
Mulanga-Kabeya *et al*, 1999 (101)	Mali, community based	Cross-sectional study, 1 months	549 pregnant women	5.0%	EIA
Bourgeois *et al*, 1998 (21)	Gabon, 3 antenatal clinics	Cross-sectional study, 5 months	646 pregnant women	9.9%	EIA
Kilmarx *et al,*1998 (69)	Thailand, antenatal clinics	Cross-sectional study, singular time point	500 pregnant mothers in Chiang Rai, 521 pregnant mothers in Bangkok	5.70% prevalence	PCR
Diallo *et al*, 1997 (38)	Ivory Coast, antenatal clinic	Cross-sectional study, 4 months	546 pregnant women	5.5%	Culture, EIA
Meda *et al*¸ 1997 (98)	Burkina Faso, 2 antenatal clinics	Cross-sectional study, duration not specified	645 pregnant women	3.1%	EIA
Joesoef *et al*, 1996 (65)	Indonesia, prenatal clinic	Cross-sectional study, 15 months	599 pregnant women	8.2%	Direct immuno-fluorescence

EIA – enzyme immunoassay, ELISA – enzyme-linked immunosorbent assay, LCR – ligase chain reaction, PCR – polymerase chain reaction

These studies diagnosed maternal infection by detecting antibodies towards *C. trachomatis* or by pathogen detection in urine samples or endocervical swabs using PCR. The median prevalence of maternal *C. trachomatis* infection is 5.80%. Particularly high prevalence of maternal *C. trachomatis* infection was identified in Vanuatu (21.5%), Mongolia (19.3%) and Tanzania (17.5%) (12,100,145).

***Group B Streptococcus.*** Twelve studies reporting the prevalence of maternal Group B *Streptococcus* (GBS) (*S. agalactiae)* colonisation were identified (**Table 3**).

**Table 3:** Characteristics and results of studies (n=12) reporting prevalence of maternal Group B *Streptococcus* (GBS) colonisation

Article	Location, setting of study	Type, duration of study	Population	Results / Prevalence	Technique used
Seoud *et al*, 2010 (134)	Lebanon, 3 hospitals	Cross-sectional study, 8 months	775 pregnant mothers	17.7% positive for GBS colonisation	Not specified
Mavenyengwa *et al*, 2010 (96)	Zimbabwe, 3 communities	Cohort study, duration not specified	780 women (one or more samples collected)	60.3% positive for GBS colonisation	Culture
Mansouri *et al*, 2008 (94)	Iran, 3 major non-private hospitals	Cross-sectional study, 11 months	602 pregnant women at childbirth	9.1% were colonised by GBS	Culture
Namavar *et aI,* 2008 (105)	Iran, hospital	Cross-sectional study, 6 months	1197 pregnant women at labour	9.1% had rectovaginal colonisation with GBS	Culture
Zusman *et al*, 2006 (168)	Brazil, 2 hospitals	Prospective study, 5 months	598 pregnant women	17.9% maternal colonisation rate	Culture
Goto *et al*, 2005 (52)	Vietnam, community based	Survey, duration not specified	505 pregnant women	4%	Culture
Larcher *et al*, 2005 (78)	Argentina, hospital	Prospective study, 18 months	1228 pregnant women	1.4% maternal colonisation rate	Culture
Sidky & Thomas, 2002 (139)	UAE, hospital	Cross-sectional study, 2 months	891 pregnant women at delivery	21.5% maternal colonisation rate	Culture
Toresani *et al*, 2001 (158)	Argentina, hospital	Cross-sectional study, 25 months	531 pregnant women	3.2% were positive for GBS	Culture
Werawatakul *et al*, 2001 (164)	Thailand, hospital	Cross-sectional study, 5 m months o	902 pregnant women presenting at labour	6.2% maternal colonisation rate	Culture
Ocampo-Torres *et al*, 2000 (111)	Mexico, 3 public hospitals	Cross-sectional study, 8 months	910 pregnant women at delivery	8.6% GBS colonisation rate	Culture, Latex agglutination
Olanisebe & Adetosoye, 1986 (113)	Nigeria, 4 government hospitals	Cross-sectional study, duration not specified	500 pregnant women (2nd and 3rd trimester)	1.6% positive for GBS	Culture

UAE – United Arab Emirates

The majority of studies diagnosed GBS colonization by direct culture of vaginal swabs. Median prevalence of maternal GBS colonization was 8.85%. The highest prevalence of maternal GBS colonization reported was 60.3% in 3 communities across Zimbabwe, which was significantly higher than the prevalence reported by other studies. However this prevalence was reported as not significantly associated with adverse perinatal outcomes (96). Higher prevalence was also noted in Lebanon and the United Arab Emirates (136,139).

***Bacterial vaginosis.*** Eleven studies reported the prevalence of bacterial vaginosis (**Table 4**).

**Table 4:** Characteristics and results of studies (n=11) reporting prevalence of bacterial vaginosis in pregnant women

Article	Location, setting of study	Type, duration of study	Population	Results / Prevalence	Technique used
Kurewa *et al*, 2010 (75)	Zimbabwe, peri-urban clinics	Cross-sectional study, 19 months	691 pregnant women	32.6%	Amsel's criteria
Msuya *et al*, 2009 (100)	Tanzania, 2 primary health clinics	Cross-sectional study, 21 months	2654 pregnant women	20.9%	Amsel's criteria
Kirakoya-Samadoulougou *et al*, 2008 (71)	Burkina Faso, 4 primary health centres	Cross-sectional study, 3 months	2133 pregnant women with analysable data	6.4%	Nugent scoring method
Romoren *et al*, 2007 (127)	Botswana, multiple antenatal clinics	Cross-sectional study, 5 months	703 pregnant women	38.0%	Microscopy
Azargoon & Darvishzadeh, 2006 (16)	Iran, hospital	Cohort study, duration not specified	1223 pregnant women	16.0%	Vaginal pH, saline wet mount, Amsel tests
Thammalangsy *et al*, 2006 (156)	Laos, 2 hospitals	Cross-sectional study, 7 months	500 pregnant antenatal attendees	14.4% by Amsel's criteria and 22.0% by Nugent's score	Amsel’s criteria, Nugent’s score
Goto *et al*, 2005 (52)	Vietnam, community based	Survey, duration not specified	505 pregnant women in 10 communes	7%	Nugent criteria
Gray *et al*, 2001 (53)	Uganda, community based	Randomised control trial, duration not specified	1576 pregnant women in the control arm of the study	48.5%	Microscopy
Mayank *et al*, 2001 (97)	India, community based	Cross-sectional study, duration not specified	600 pregnant women	18%	Microscopy
Taha *et al*, 1999 (150)	Malawi, hospital	Cross-sectional study, 25 months	9126 pregnant women	30%	Vaginal wet mounts
Meda *et al*, 1997 (98)	Burkina Faso, 2 antenatal clinics	Cross-sectional study, duration not specified	645 pregnant women	13%	Microscopy

The median prevalence of maternal bacterial vaginosis was 20.9%. Especially high prevalence was reported in Uganda, Botswana and Zimbabwe, highlighting high prevalence of bacterial vaginosis in sub-Saharan Africa. The majority of studies used microscopy of vaginal wet mounts in combination with established criteria for diagnosing bacterial vaginosis (53,75,126).

### Prevalence of Viral Pathogens

**Figure 10:**
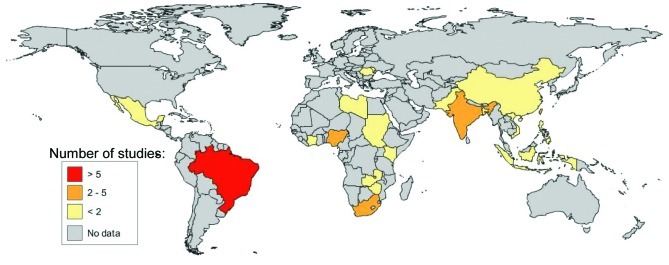
Geographical distribution of studies (n=39) providing information on prevalence of maternal hepatitis B virus (HBV) infection; “no data” in the legend refers to low and middle-income countries only, as data from high-income countries were not the subject of this study.

**Figure 11:**
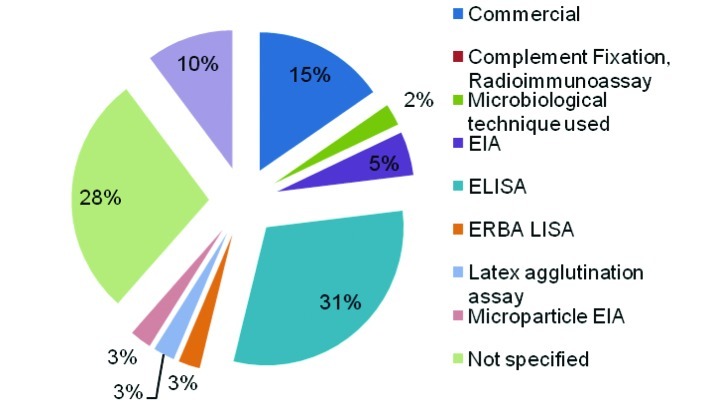
Techniques used to identify hepatitis B virus (HBV) infection in 39 studies.

**Figure 12:**
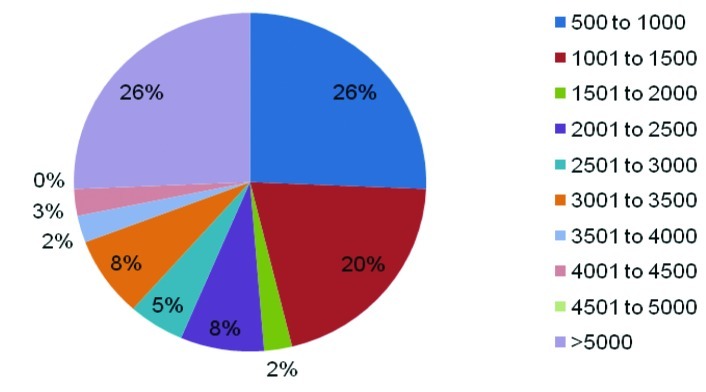
Sizes of study populations in 39 studies reporting maternal hepatitis B (HBV) infection prevalence.

**Figure 13:**
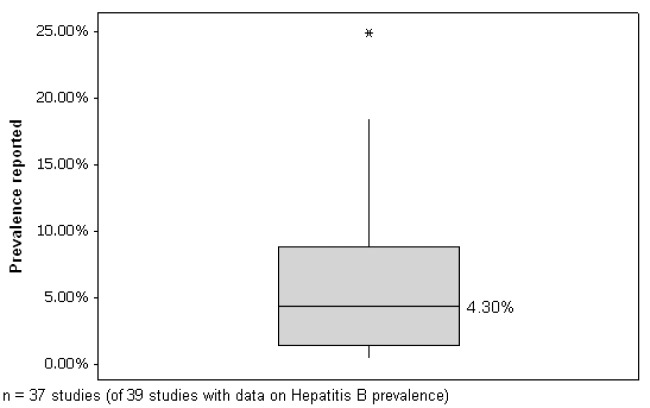
Box plot showing prevalence of hepatitis B virus (HBV) infection detected in relevant studies (n=37). Only 30 of 37 relevant studies measured prevalence by detecting Hepatitis B surface antigen (HBsAg) in pregnant women. The following number summaries are depicted in the boxplot: the smallest observation (sample minimum), lower quartile (25%), median (50%), upper quartile (75%), and largest observation (sample maximum). Asterisk indicates an outlier.

***Hepatitis B virus.*** Thirty-nine studies characterizing the prevalence of maternal Hepatitis B infection were identified (**Supplementary Table 3**)(Supplementary Table 3), and their features and results are summarized in **Figures 10**,******11**,******12******and******13**.

The majority of identified studies were conducted in a health care facility (87.2%) whilst 5.1% were community based and 7.7% of studies did not specify the setting. Most of the studies were also cross-sectional (69.2%) in nature, with remaining studies being retrospective observational (17.9%), surveys (5.1%) and either prospective, cohort or case-control studies (2.5% each).

The majority of studies screened for the presence of maternal HBV infection by detecting Hepatitis B surface antigen (HBsAg) in maternal serum. Particularly high maternal HBV prevalence (25%) was identified in Zimbabwe (90), Brazil (20) and Taiwan (83).

**Figure 14:**
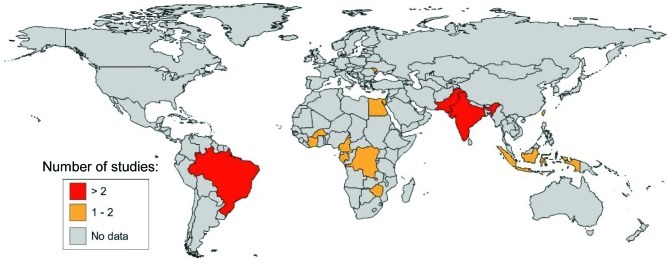
Geographical distribution of studies (n=21) providing reporting prevalence of maternal hepatitis C (HCV) infection; “no data” in the legend refers to low and middle-income countries only, as data from high-income countries were not the subject of this study.

**Figure 15:**
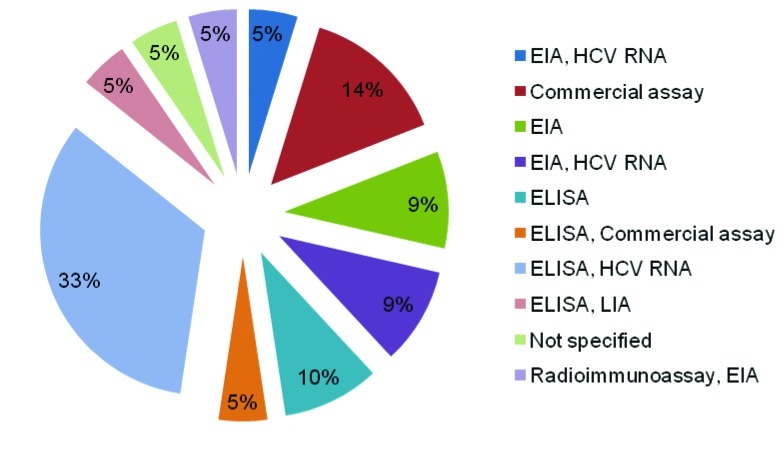
Techniques used to diagnose maternal hepatitis C (HCV) infection (n=21 studies); HCV RNA refers to tests detecting HCV RNA, including PCR and RT-PCR.

**Figure 16:**
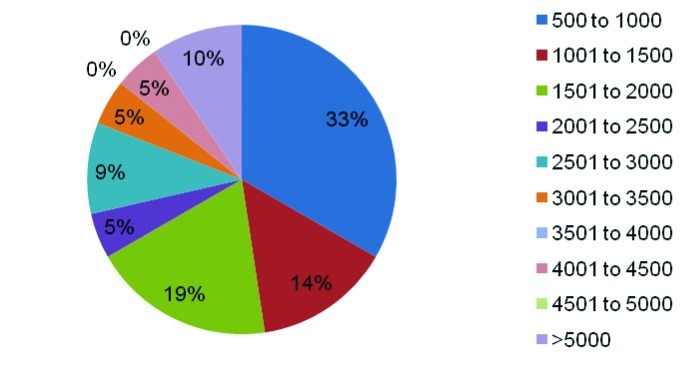
Size of study populations of 21 studies reporting maternal hepatitis C (HCV) infection prevalence.

**Figure 17:**
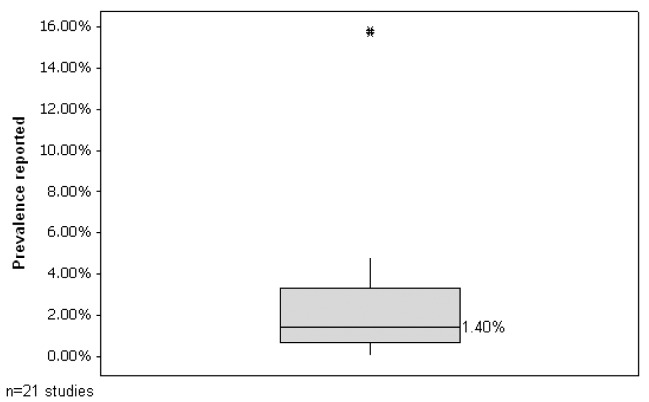
Box plot showing prevalence of hepatitis C virus (HCV) exposure detected in relevant studies (n=21). All studies diagnosed the history of HCV infection by detecting anti-HCV antibodies in serum. The following number summaries are depicted in the boxplot: the smallest observation (sample minimum), lower quartile (25%), median (50%), upper quartile (75%), and largest observation (sample maximum). Asterisk indicates an outlier.

***Hepatitis C virus.*** Twenty-one studies reporting the prevalence of maternal Hepatitis C virus (HCV) infection were identified (**Supplementary Table 4**)(Supplementary Table 4). The features and findings of these studies are summarized in **Figures 14**,******15**,******16******and **17**.

Almost all studies reporting maternal HCV prevalence were conducted in health care facilities (95.2%) and one did not specify the study setting. The majority of studies were also cross-sectional (80.9%), with the remaining studies being case-control studies (14.3%), prospective studies (9.5%) and one serological survey (4.8%).

Median maternal exposure to HCV (anti-HCV) prevalence reported was 1.4%. Active infection prevalence (HCV RNA) was reported in 6 studies and median active HCV infection prevalence from these studies was 1.2%. Two studies from Egypt reported especially high prevalence of maternal HCV exposure (15.8% and 15.7%) and active infection rates (10.8% and 10.9%), highlighting a local problem with maternal HCV infection in Egypt (136,143).

***Rubella virus.*** Fifteen studies characterizing the epidemiology of maternal rubella were identified (**Table 5**).

**Table 5:** Characteristics and results of studies (n=15) reporting prevalence of maternal *Rubella* infection

Article	Location, setting of study	Type, duration of study	Population	Results / Prevalence	Technique used
Lin *et al*, 2010 (86)	Taiwan, hospital	Cross-sectional study, 7 years	10 089 pregnant women	Seronegativity was 14.0%	Microparticle EIA
Tamer *et al*, 2009 (152)	Turkey, antenatal clinic	Cross-sectional study, duration not specified	1972 serum samples from pregnant women	Seropositivity for anti-rubella IgG, IgM and IgG+IgM together was 96.1%, 0.2% and 1.8%, respectively	Commercial ELISA (detecting IgG and IgM)
Ai & Ee, 2008 (8)	Malaysia, antenatal clinics and hospital	Cross-sectional study, duration not specified	500 pregnant mothers	11.4% were susceptible to Rubella	Rubella IgG studies
Majlessi *et al*, 2008 (92)	Iran, health centres	Cross-sectional study, 2 years	965 pregnant women	Estimated rubella immunity rate was 91.1%, Nonimmunity rate was 8.9%	ELISA
Das *et al*, 2007 (34)	India, hospital	Screening, duration not specified	1115 pregnant women with bad obstetric history, 500 normal pregnant women	3.6% seropositivity (*BOH), 0% seropositivity (normal)	ELISA (Detecting IgM)
Ocak *et al*, 2007 (110)	Turkey, antenatal clinic	Retrospective observational, 23 months	1652 pregnant women	Anti-rubella IgG and IgM antibodies were reactive in 95.0%, and in 0.54%	ELISA (detecting IgG and IgM)
Pehlivan *et al*, 2007 (119)	Turkey, community based	Cross-sectional study, 7 mo months	824 women from 60 clusters; 803 eligible for serological study	93.8% positive for anti-rubella IgG, 0.6% were IgM and IgG positive, 5.6% were susceptible	Micro ELISA (detecting IgG and IgM)
Tseng *et al*, 2006 (160)	Taiwan, hospital	Retrospective observational, 4 years	5007 pregnant women	13.4% susceptible among Taiwanese women; 29.1% susceptible among non-Taiwanese women	Microparticle EIA
Barreto *et al*, 2006 (18)	Mozambique, antenatal clinics	Cross-sectional serosurvey, 3 months	974 pregnant women at antenatal clinic attendance	95.3% positive for Rubella IgG	ELISA
Corcoran & Hardie, 2006 (31)	South Africa, antenatal clinics	Cross-sectional study, duration not specified	1200 serum samples from a 2003 HIV/syphilis survey	96.5% immune	ELISA
Desinor *et al*, 2004 (36)	Haiti, hospital	Cross-sectional study, 4 months	503 pregnant women; 8 excluded leaving 495	95.2% were seropositive	EIA
Weerasekera *et al,* 2003 (163)	Sri Lanka, antenatal clinic	Cross-sectional study, 2 years	500 maternal blood samples, before 16th week of gestation	82% were positive for rubella specific IgG, 75% gave a history of vaccination against rubella before their present pregnancy	ELISA (detecting IgG and IgM)
Palihawadana *et al*, 2003 (116)	Sri Lanka, multiple antenatal clinics	Cross-sectional study, duration not specified	620 pregnant women	76% of pregnant females were seropositive	ELISA (detecting IgG)
Ashrafunnessa Khatun, *et al*, 2000 (15)	Bangladesh, hospital	Cross-sectional study, 11 months	609 pregnant women	85.9% were seropositive and 14.1% were seronegative	ELISA
Dos Santos *et al*, 2005 (39)	Brazil, prenatal testing	Cross-sectional study, 8 months	1024 pregnant women	77.4%	Haemagglutinin Inhibition Assay

EIA – enzyme immunoassay, ELISA – enzyme-linked immunosorbent assay

These studies detected the presence of maternal anti-rubella IgG as a marker of past infection or immunization and mothers who did not possess these antibodies were susceptible to rubella infection. Maternal IgM was detected in some studies as a marker of recent or current infection, which is associated with an increased risk of vertical transmission. Median maternal susceptibility to rubella was 8.9%. Higher susceptibility rates were reported in Sri Lanka, Brazil and Taiwan (2 studies) (39,84,160,163).

***Cytomegalovirus.*** Five studies on maternal cytomegalovirus (CMV) infection prevalence were identified (**Table 6**).

**Table 6:** Characteristics and results of studies (n=5) reporting prevalence of maternal cytomegalovirus (CMV) infection

Article	Location, setting of study	Type, duration of study	Population	Results / Prevalence	Technique used
Tabatabaee *et al*, 2009 (149)	Iran, hospital	Cross-sectional study, 7 months	1472 pregnant women presenting at labour	97.69% seropositivity, 2.31% seronegativity; prevalence of active infection – 4.35%	Not specified
Das *et al*, 2007 (34)	India, hospital	Cross-sectional study	1115 pregnant women with Bad obstetric history, 500 normal pregnant women	11% prevalence in women with Bad obstetric history, 4% prevalence in normal pregnant women	Commercial ELISA kit detecting anti-CMV IgM
Ocak *et al*, 2007 (110)	Turkey, hospital	Retrospective observational study, 2 years	1652 pregnant women	94.9%seropositivity for anti-CMV IgG, 0.4%positive for anti-CMV IgM	ELISA detecting anti-CMV IgG and IgM
Suarez *et al*, 1994 (144)	Chile, public outpatient department and a special clinic for university students	Cross-sectional study, 3 years	939 pregnant women of a low socioeconomic level, and 123 pregnant university students	95% in low socioeconomic class; 69.9% in pregnant students; 2 primary infections occurred (1 in each group)	ELISA; initially seronegative women were tested again during 2^nd^ and 3^rd^ trimester to identify primary infections
Tamer *et al*, 2009 (152)	Turkey, antenatal clinics	Cross-sectional study, singular time point	1972 samples of sera from pregnant women	Seroprevalence of anti-CMV IgG, IgM and IgG+IgM together were found in 96.4%, 0.7% and 1.9% of the pregnant women, respectively	Commercial ELISA kit

ELISA – enzyme-linked immunosorbent assay

The median prevalence of maternal IgG to CMV (calculated from 4 studies that reported this) was 95.7%, indicating a high proportion of mothers with previous exposure to CMV. One hospital-based study in India identified a statistically significant higher prevalence of CMV IgM (indicating active or recent infection) in mothers with Bad Obstetric History (BOH), highlighting a role for maternal CMV infection in adverse pregnancy outcome in this setting (34).

***Herpes simplex virus.*** Five studies outlining the prevalence of maternal Herpes simplex virus 2 (HSV-2) were identified (**Table 7**).

**Table 7:** Characteristics and results of studies (n=5) reporting prevalence of maternal Herpes simplex virus (HSV) infection

Article	Location, setting of study	Type, duration of study	Population	Results / Prevalence	Technique used
Kurewa *et al*, 2010 (75)	Zimbabwe, peri-urban clinics	Cross-sectional study, 19 months	691 pregnant women	51.10% seropositive	ELISA detecting IgG
Yahya-Malima *et al*,2008 (166)	Tanzania, antenatal clinics (6)	Cross-sectional study, duration not specified	1296 sera collected from pregnant women	20.7% prevalence of genital herpes	ELISA
Chen *et al*, 2007 (28)	China, antenatal clinic	Cross-sectional study, 3 months	502 pregnant women	10.8% seroprevalence of HSV-2	Commercial ELISA to detect IgG
Haddow *et al*, 2007 (56)	Vanuatu, antenatal clinic	Cross-sectional study, 1 to 2 years	535 pregnant women	32% seroprevalence of HSV-2	ELISA
Joesoef *et al*, 1996 (65)	Indonesia, prenatal clinic	Cross-sectional study, 15 months	599 pregnant women	9.9% seroprevalence of HSV-2	Immuno-blot

ELISA – enzyme-linked immunosorbent assay

These studies detected the presence of antibodies to HSV as a marker of maternal infection. Median prevalence of HSV-2 was 20.7%. Higher seroprevalences were noted in Zimbabwe, Vanuatu and Tanzania (56,75,166).

## DISCUSSION

### Prevalence of bacterial and viral maternal infections

Our search of published literature relevant to the aetiology and epidemiology of bacterial and viral maternal infections in the developing world retrieved 499 titles. Analysis of these titles yielded 158 studies which provided detailed epidemiological information on 10 maternal infections. The 5 bacterial and 5 viral maternal infections identified in this panel represent maternal infections that were most extensively studied, suggesting that these infections have a high burden on pregnancy outcomes in the developing world. These infections also have potential adverse effects on neonates.

Our review confirms the suspected high prevalence of bacterial and viral maternal infections in the developing world, as demonstrated by the median prevalence rates calculated for each pathogen studied. Of particular concern are the high prevalence rates of maternal syphilis (2.6%), *C. trachomatis* (5.8%), bacterial vaginosis (20.9%), hepatitis B virus (4.3%) and *Herpes simplex* virus (20.7%).

The prevalence of these infections also showed significant variance between countries and regions. The prevalence of maternal infections in sub-Saharan Africa is especially high, specifically in Zimbabwe (75,79,96,130), Tanzania (101,166) and Cameroon (66,107). Previous studies have shown that all-cause obstetric risk and maternal mortality ratio are highest in Sub-Saharan Africa (1). The high prevalence of maternal infections in this region may have an important contributory role towards the high maternal morbidity and mortality seen in Sub-Saharan Africa. Regional differences in the prevalence of maternal infections are likely to be closely related to the quality of reproductive health care available in different regions, or unique local scenarios.

### Gaps in existing knowledge

In the process of reviewing the subject, we identified several facility-based retrospective studies reporting causes of maternal mortality. Many of these studies attributed a proportion of deaths to infection or sepsis, but were unable to provide microbiological or serological evidence of the specific aetiology of infection. Thus, these studies had to be excluded from the final panel of studies that we reviewed. This highlights a gap in existing knowledge on the epidemiology and impact of maternal infection, especially on the aetiology of infectious agents that lead to puerperal sepsis and subsequent mortality. Increased surveillance and diagnostic capabilities in health care facilities and in the community is needed to identify the aetiological agents responsible for puerperal sepsis and maternal mortality.

The prevalence of maternal infection reported by the studies identified in this review may be an underestimate of actual rates of infection as not all pregnant women in developing countries may have access to or choose to access formalized antenatal care. This could be due to financial constraints, difficulties in accessing these facilities and personal or cultural beliefs. In addition, antenatal care services may not have the capacity to routinely screen for maternal infections, especially those that are asymptomatic (such as *N. gonorrhoeae* and *C. trachomatis*) and those that require serological tests such as PCR and ELISA to diagnose (Hepatitis B and C), due to limited resources or expertise. These infrastructural problems are essential contributors to the persistence of high maternal morbidity and mortality in developing countries and need to be overcome in order to accurately characterize the burden of maternal infections in these countries.

### Strengths and limitations

This is one of the first reviews to summarise the epidemiology of bacterial and viral maternal infections in the developing world (7). The search strategy devised is sensitive and specific, which allowed for a comprehensive review of available literature on this topic. The information generated in this review can be utilised to guide public health policy and the allocation of resources within local governments and by the international community towards improving maternal health. Limitations of this work include the exclusion of studies with less than 500 participants and the omission of pathogens with less than 5 papers reporting their prevalence. This was done to minimise the potential confounding effect that smaller, underpowered studies may have had on the overall prevalences reported and to increase the statistical robustness of the data presented.

This study could be further improved by analysing smaller studies that were identified and performing a sensitivity analysis of their results prior to inclusion. Also, it is likely that further valuable insights may be obtained from non-English articles of studies conducted in francophone parts of Africa (in French), South America (in Spanish) and in China (in Chinese), which could be accessed from appropriate databases. Reviewing non-English articles may assist in defining the epidemiology of pathogens for which we managed to identify few (<5) studies, as well as providing more robust data on the pathogens presented in this review. In addition, searching grey (unpublished) literature or contacting health officials and researchers in the field may also yield more country specific data on the subject, thus enabling more targeted and context-specific public health measures.

### Recommendations and future work

Reducing the prevalence of maternal infections, and consequently maternal and neonatal morbidity and mortality, requires a concerted, multifaceted approach. Improvements in the provision, accessibility and uptake of antenatal care services are absolutely essential to reduce the prevalence of not only maternal infection but also other causes of maternal morbidity and mortality. This entails an improvement in antenatal booking, the number of antenatal visits and childbearing with professional assistance (40). Wherever possible, routine screening and treatment for maternal infections should be conducted. Alternatives to antenatal screening include syndromic management or risk assessment based approaches to treat maternal infection. Routine immunisation against vaccine-preventable diseases should also be implemented to reduce the eventual burden that these infections may have on pregnancy outcomes and neonates (7,132).

We hope that the gaps in information highlighted in this study will guide the design and implementation of studies to accurately assess the epidemiology of maternal infections in the developing world, especially in countries where the prevalence of maternal infection is unreported. Ideally, studies should be large, community-based and longitudinal, and investigate the association between pregnancy outcome and microbiological and serological evidence of maternal infection to accurately define the burden of maternal infections and their impact on pregnancy outcome (132). There is also a great need for the design of rapid point-of-care diagnostic tests for use in the field for the diagnosis of maternal infections. Affordable and novel therapeutics and interventions will also be beneficial in reducing the impact of maternal infections. These measures are dependent upon the co-operation of the research community and the altruism of industry to succeed.

More than US$ 40 billion (€ 30 billion) has been pledged towards the newly formed Global Strategy for Women’s and Children’s Health (169). These funds should be spent prudently on effective and sustainable measures to improve maternal health. The majority of this allocation should go towards the strengthening of basic antenatal care systems in developing countries. Because serious maternal infections are a major contributor to maternal morbidity and mortality, the early detection and treatment of infections is an important component of prenatal care. The continued support of the global community is also needed to ensure the improvement of maternal health in the developing world.

### Conclusion

This review highlights the high bacterial and viral maternal infection rates in the developing world. Urgent, concerted action is required to reduce the burden of these infections. In addition to raising awareness about the severity of the problem of maternal infections in the developing world, data from this review will be beneficial in guiding public health policy, research interests and donor funding towards achieving MDG 5.
