# Supplementary material for: Epidemiology and aetiology of maternal bacterial and viral infections in low- and middle-income countries
Source: J Glob Health. 2011 Dec;1(2):171–88. (PMC3484781)
Supplement: Supplementary Table 1 [file jogh-01-171-s001.pdf]

Supplementary table 1. Summary of data extracted from studies (n=72) reporting prevalence of maternal syphilis infection

Author, Year of publication	Country, Setting of Study	Number studied	Prevalence	Study design	Duration of study	Technique used
Kurewa *et al*, 2010	Zimbabwe, peri-urban clinics	691	1.20%	Cross sectional	19m	RPR, TPHA
Bukar *et al*, 2009	Nigeria, hospital	18101	0.05%	Retrospective observational	10y	VDRL, TPHA
De Lima *et al*, 2009	Brazil, hospital	534	2.70%	Cross sectional	9m	VDRL
Inagaki *et al*, 2009	Brazil, ANC and hospital	9550	0.90%	Cross sectional	NS	NS
Lopez-Zambrano *et al*, 2009	Venezuela, ANC	140336	0.81%	Retrospective observational	5y	VDRL
Msuya *et al*, 2009	Tanzania, primary healthcare clinics	2654	0.90%	Cross sectional	21m	RPR, Commercial assay
Cheng *et al*, 2008	China, hospital	418871	0.48%	Questionnaire and screening	2y	TRUST, TPPA
Kinoshita-moleka *et al*, 2008	Democratic Republic of Congo, maternity clinics	529	0.00%	Cross sectional	4m	RPR, TPHA
Kizito *et al*, 2008	Uganda, hospital	17047	4.00%	Screening	NS	NS
Kwiek *et al*, 2008	Malawi, hospital	3824	5.00%	Cross sectional	4y	RPR, TPHA
Lujan *et al*, 2008	Mozambique, ANC	1119	4.70%	Cross sectional	5m	RPR, TPHA
Plewes *et al*, 2008	Thailand and Burma, ANC	741	0.40%	Screening	NS	NS
Todd *et al*, 2008	Afghanistan, hospital	4452	0.00%	Cross sectional	4m	Commercial assay
Yahya-Malima *et al*, 2008	Tanzania, ANC	1296	1.60%	Cross sectional	NS	RPR, TPHA
Bronzan *et al*, 2007	South Africa, ANC	1250	6.30%	Screening	NS	RPR, TPHA, ICS
Cheng *et al*, 2007	China, hospitals (61)	477656	0.50%	Screening	2.5y	TRUST, TPPA
Garcia *et al*, 2007	Bolivia, hospital and ANC	11618	5.00%	Cross sectional	NS	Commercial assay
Revollo *et al*, 2007	Bolivia, hospital	1594	7.20%	Cross sectional	5m	Commercial assay, RPR
Sethi *et al*, 2007	India, ANC	40511	1.82%	Retrospective observational	10y	VDRL, TPPA
Taiwo *et al*, 2007	Nigeria, hospital	505	2.97%	Screening	NS	RPR, TPHA
Chen *et al*, 2006	China, ANC	504	0.20%	Cross sectional	3m	RPR, TPHA
Devjee *et al*, 2006	South Africa, ANC	1856	3.90%	Cross sectional	NS	RPR, TPHA
Hernandez-Trejo *et al*, 2006	Mexico, hospital	1322	0.30%	Cross sectional	NS	Commercial assay, VDRL, FTA-ABS
Hoekstra *et al*, 2006	Nicaragua, ANC	1059	1.50%	Screening	3m	ELISA, VDRL, TPHA
Ikeme &	Nigeria, hospital	7175	0.13%	Retrospective	5y	VDRL

Okeke, 2006				observational		
Munkhuu *et al*, 2006	Mongolia, ANC	2735	2.00%	Retrospective observational	NS	NS
Swai *et al*, 2006	Tanzania, ANC	17323	7.30%	Screening	12m	RPR
Pankratov *et al*, 2006	Belarus, not specified	NS	10.50%	Retrospective observational	9y	Dark ground microscopy, FTA-ABS
Amindavaa *et al*, 2005	Mongolia, prenatal clinic	2000	6.40%	Cross sectional	11m	Commercial assay, TPHA, FTA-ABS
Creek *et al*, 2005	Botswana, ANC	750	4.30%	Rstrospective observational	10y	NS
Goto *et al*, 2005	Vietnam, community-based	505	0.00%	Survey	NS	RPR, TPHA
Pham *et al*, 2005	Zimbabwe, hospital	2969	2.50%	Cross sectional	NS	RPR
Rey *et al*, 2005	Ivory Coast, hospital	4320	1.40%	Screening	18m	VDRL, TPHA
Apea-Kubi *et al*, 2004	Ghana, hospital	517	5.60%	Cross sectional	NS	TPPA
Gichangi *et al*, 2004	Kenya, hospital	12414	3.00%	Cross sectional	NS	RPR
Labbe *et al*, 2004	Guinea-Bissau, hospital	743	3.90%	Case control	12m	RPR
Myer *et al*, 2004	South Africa, ANC	8917	12.00%	Screening	NS	RPR
Rodrigues & Guimaraes, 2004	Brazil, health centres	3047	1.70%	Cross sectional	NS	VDRL, FTA-ABS
Majoko *et al*, 2003	Zimbabwe, rural health centres	12095	11.40%	Cross sectional	3y	RPR
Sullivan *et al*, 2003	Vanuatu, ANC	547	2.40%	Cross sectional	12m	RPR
Tsegaye *et al*, 2003	Ethiopia, ANC	1183	2.20%	Cross sectional	6y	RPR, TPHA
Watson-Jones *et al*, 2002	Tanzania, hospital	1809	8.00%	Cohort	2y	RPR
Mathai *et al*, 2001	India, ANC	7950	0.98%	Retrospective observational	12m	VDRL
Gray *et al*, 2001	Uganda, community-based	1376	3.30%	Randomised control trial	NS	TRUST, TPHA, FTA-ABS
Mayank *et al*, 2001	India, community-based	600	11.00%	Cross sectional	NS	VDRL, TPHA
Southwick *et al*, 2001	Bolivia, hospital	1428	4.30%	Cross sectional	5m	RPR
Rotchford *et al*, 2000	South Africa, ANC	1783	9.00%	Cross sectional	5m	RPR
Temmerman *et al*, 2000	Kenya, hospital	12414	2.38%	Cross sectional	12m	RPR, TPHA
Barsanti *et al*, 1999	Brazil, hospital	1000	2.40%	Cross sectional	12m	VDRL, TPHA, ELISA
Mulanga-Kabeya *et al*, 1999	Mali, community-based	549	2.00%	Cross sectional	1m	RPR, TPHA
Ozumba *et al*, 1999	Nigeria, hospital	11428	1.30%	Retrospective observational	7y	RPR, TPHA
Temmerman	Kenya, clinics	81311	5.30%	Retrospective	3y	RPR

et al, 1999				observational		
Bourgeois *et al*, 1998	Gabon, ANC	646	2.90%	Cross sectional	5m	RPR
Keou *et al*, 1998	Cameroon, not specified	4100	17.40%	NS	24m	NS
Kilmarx *et al*, 1998	Thailand, ANC	1021	0.50%	Cross sectional	NS	VDRL, RPR
Diallo *et al*, 1997	Ivory Coast, ANC	546	1.10%	Cross sectional	4m	RPR, TPHA
Meda *et al*, 1997	Burkina Faso, ANC	645	3.60%	Cross sectional	NS	RPR, TPHA
Sangare *et al*, 1997	Burkina Faso, ANC	1294	2.50%	Cross sectional	5m	RPR, TPHA
Amaral *et al*, 1996	Brazil, hospital	5815	1.16%	Cross sectional	6m	VDRL, TPHA
Joesoef *et al*, 1996	Indonesia, prenatal clinic	599	0.70%	Cross sectonal	15m	TRUST
Mwakagile *et al*, 1996	Tanzania, ANC	777	4.00%	Cross sectional	NS	NS
dos Santos *et al,* 1995	Brazil, prenatal clinic	1024	3.91%	Cross sectional	8m	VDRL, FTA-ABS
Jenniskens *et al*, 1995	Kenya, ANC	13131	6.50%	Screening	12m	RPR
Bam, 1994	South Africa, hospital	1476	15.00%	Cross sectional	NS	RPR, VDRL, TPHA
Rutgers, 1993	Zimbabwe, ANC	1433	14.00%	Cross sectional	12m	RPR
Chang *et al*, 1992	Taiwan, prenatal clinic	1955	0.97%	Screening	3.5y	NS
Temmerman *et al*, 1992	Kenya, health centres	488	3.60%	Prospective	24m	RPR, TPHA
Greenwood *et al*, 1992	Gambia, Not specified	826	9.30%	Cross sectional	NS	RPR, TPHA
Ndumbe *et al*, 1992	Cameroon, ANC	544	15.90%	Cross sectional	NS	Commercial assay
Gini *et al*, 1989	Nigeria, ANC	29083	0.35%	Screening	5y	VDRL, TPHA
Goh & Ngeow, 1989	Malaysia, ANC	14841	1.05%	Screening	NS	VDRL, TPHA
Liljestrand *et al,* 1985	Mozambique, ANC	1468	6.34%	Cross sectional	12m	VDRL, TPHA, FTA-ABS

ANC – antenatal clinic; m – month; y – year; NS – not stated; RPR – rapid plasma regain; TPHA – Treponema pallidum haemagglutination; VDRL – Venereal Disease Research Laboratory; TRUST – toluidine red unheated serum test; TPPA – Treponema pallidum particle agglutination; ICS – immunochromatographic strip; FTA-ABS – fluorescent treponemal antibody-absorption;

Supplementary table 2. Summary of data extracted from studies (n=21) reporting prevalence of maternal *Neisseria gonorrhoeae* (NG) infection

Author, Year of publication	Country, Setting of Study	Number studied	Prevalence	Study design	Duration of study	Technique used
Jalil *et al*, 2008	Brazil, prenatal services	3003	1.50%	Cross sectional	12m	Hybrid capture technique
Romoren *et al*, 2007	Botswana, ANC	703	3.00%	Cross sectional	NS	LCR
Apea-Kubi *et al*, 2004	Ghana, hospital	517	0.60%	Cross sectional	NS	RNA detection kit
Latif *et al*, 1999	Zimbabwe, ANC and primary care clinics	1189	5.80%	Cross sectional	NS	NS
Kilmarx *et al*, 1998	Thailand, ANC	1021	0.20%	Cross sectional	NS	PCR
Msuya *et al*, 2009	Tanzania, primary health clinics	2654	0.50%	Cross sectional	21m	Culture for NG
Kinoshita-moleka *et al*, 2008	Democratic Republic of Congo, maternity clinics	529	0.40%	Cross sectional	4m	PCR
Lujan *et al*, 2008	Mozambique, ANC	835	2.50%	Cross sectional	5m	PCR
Thammalangsy *et al*, 2006	Laos, hospital	500	0.80%	Cross sectional	7m	PCR
Chen *et al*, 2006	China, ANC	504	0.80%	Cross sectional	3m	PCR
Goto *et al*, 2005	Vietnam, community-based	505	0.00%	Cross sectional	NS	Culture
Amindavaa *et al*, 2005	Mongolia, prenatal clinic	2000	6.10%	Cross sectional	11m	PCR
Mayank *et al*, 2001	India, community-based	600	0.30%	Cross sectional	NS	Culture
Gray *et al*, 2001	Uganda, community-based	1394	1.70%	RCT	NS	LCR
Sullivan *et al*, 2003	Vanuatu, ANC	547	5.90%	Cross sectional	12m	PCR
Diallo *et al*, 1997	Ivory Coast, ANC	546	3.70%	Cross sectional	4m	Culture
Meda *et al*, 1997	Burkina Faso, ANC	645	1.60%	Cross sectional	NS	Culture
Mwakagile *et al*, 1996	Tanzania, ANC	777	3.60%	Cross sectional	NS	Microscopy
Joesoef *et al*, 1996	Indonesia, prenatal clinic	599	0.80%	Cross sectional	15m	Culture
Mulanga-Kabeya *et al*, 1999	Mali, community-based	549	1.00%	Cross sectional	1m	Culture
Bourgeois *et al*, 1998	Gabon, ANC	646	1.90%	Cross sectional	5m	Culture

m – month; y – year; ANC – antenatal clinic; LCR – ligase chain reaction; PCR NS – not stated; RCT – randomised control trial

Supplementary table 3. Summary of data extracted from studies (n=39) reporting prevalence of maternal Hepatitis B virus infection

Author, Year of publication	Country, Setting of Study	Number studied	Prevalence *	Study design	Duration of study	Technique used
El-Magrahe *et al*, 2010	Libya, hospital	1500	1.50%	Cross sectional	NS	ELISA
Zhang *et al*, 2010	China, not specified	6398	6.71%	Cross sectional	23m	NS
Chatterjee *et al*, 2009	India, prenatal clinics	36379	0.82%	Cross sectional	2y 9m	ELISA, Commercial assay
De Lima & Viana, 2009	Brazil, hospital	534	1.10%	Cross sectional	9m	NS
Evelyn *et al*, 2009	Nigeria, ANC	3465	1.60%	Cross sectional/cohort study	3y	Latex agglutination assay
Sheikh, 2009	Pakistan, hospital	2592	0.34%	Cross sectional	1y	Commercial assay
Shrestha *et al,* 2009	Nepal, hospital	5602	3.21%†	Retrospective observational	6y	NS
Lin *et al*, 2008	Taiwan, hospital	10327	15.50%	Retrospective observational	5y	Microparticle EIA
Miyamoto & Bertolini, 2008	Brazil, hospital	12274	1.00%	Retrospective observational	5y	Commercial assay
Singla & Chander, 2008	India, ANC	2933	1.73%	Retrospective observational	4y	ELISA
Todd *et ali,* 2008	Afghanistan, hospital	4452	1.53%	Cross sectional	4m	Commercial assay
Elsheikh *et al*, 2007	Sudan, hospital	728	5.60%	Cross sectional	3m	ELISA
Burnett *et al*, 2007	South Africa, ANC	710	2.2%†	Retrospective case control	3m (over 3 years)	Commercial assay
Liu *et al*, 2007	Taiwan, community-based	16926	10.80%	Retrospective observational	3y	NS
Okoth *et al*, 2006	Kenya, ANC	2241	9.30%	Cross sectional observational	1y	NS
Bertolini *et al*, 2006	Brazil, prenatal service	3188	18.50%	Cross sectional	4y	ELISA
Ikeme *et al*, 2006	Nigeria, ANC	7581	0.84%	Cross sectional/screening	5y	NS
Obi *et al*, 2006	Nigeria, ANC	1499	4.60%	Prospective case-control	8m	NS
Akani *et al*, 2005	Nigeria, AND	600	4.30%	Cross sectional	NS	ELISA
Goto *et al*, 2005	Vietnam, community-based	505	10.00%	Survey	NS	ELISA
Surya *et al*, 2005	Indonesia, hospital	2450	1.90%	Cross sectional	5m	Commercial assay
Rouet *et al*, 2004	Ivory Coast, women recruited for clinical trial	501	8.00%	Case control	NS	NS
Vazquez-Martinez *et al*, 2003	Mexico, ANC	9992	1.65%	Cross sectional	4m	ELISA
Lin *et al,* 2003	Taiwan, not specified	3605	12.00%	Retrospective observational	15y	Radioimmunoassay

Lewis-Ximenez *et al*, 2002	Brazil, hospital	874	0.50%	Cross sectional	2m	NS
Drobeniuc *et al*, 1999	Moldova, prenatal clinic	1098	9.70%	Cross sectional	NS	Radioimmu-noassay
Madzime *et al*, 1999	Zimbabwe, hospital	984	25.00%	serological survey	12m	EIA
Prakash *et al*, 1998	India, ANC	1112	9.50%	Cross sectional	NS	ELISA
Duarte *et al*, 1997	Brazil, hospital	7992	0.95%	Cross sectional	2y 10m	ELISA
dos Santos *et al*, 1995	Brazil, prenatal clinic	1024	0.60%	Cross sectional	8m	ELISA
Gill *et al*, 1995	India, ANC	2000	5.00%	Cross sectional	NS	ELISA
Oshitani *et al*, 1995	Zambia, ANC	2098	6.50%	Cross sectional	NS	NS
Woodruff *et al*, 1993	Romania, prenatal clinics	573	8.38%	Cross sectional	1m	Radioimmu-noassay
Guidozzi *et al*, 1993	South Africa, not specified	3469	1.21%	Cross sectional	NS	NS
Akhter *et al*, 1992	Bangladesh, hospital	500	3.60%	Cross sectional	3m	Commercial assay
Sebastian *et al*, 1990	Brunei, ANC	1267	3.20%	Cross sectional	NS	ELISA
Kew *et al*, 1987	South Africa, ANC	1234	2.61%	Cohort	NS	Radioimmu-noassay
Sy *et al*, 1986	Philippines, prenatal clinic	5684	7.60%	Cross sectional	17m	EIA
Anderson *et al*, 1975	Taiwan, prenatal clinic	1106	7.50%	Cross sectional	12m	Complement Fixation, Radioimmu-noassay

m – month; y – year; ANC – antenatal clinic; ELISA – enzyme-linked immunosorbent assay; EIA – enzyme immunosorbent assay; NS – not stated

*Reported as number of pregnant women who possessed Hepatits B surface Antigen (HBsAg).

†These two studies used detection of maternal HBV DNA to describe prevalence.

Supplementary table 4. Summary of data extracted from studies (n=21) reporting prevalence of maternal Hepatitis C virus infection

Author, Year of publication	Country, Setting of Study	Number studied	Prevalence *	Study design	Duration of study	Technique used
Costa *et al*, 2009	Brazil, ANC	28,561	0.15%	Cross sectional	1 -2 yrs	EIA, HCV RNA†
De Lima & Viana, 2009	Brazil, hospital	534	1.40%	Cross sectional	9m	NS
Parthiban *et al*, 2009	India, ANC	3115	0.60%	Prospective	29m	ELISA, RT-PCR
Shebl *et al*, 2009	Egypt, community health unit	1,863	15.70%	Prospective cohort	NS	EIA, RT-PCR
Sheikh, 2009	Pakistan, hospital	2,592	0.69%	Cross sectional	1y	Commercial assay
Ndong-Atome *et al*, 2008	Gabon, ANC	947	2.10%	Cross sectional	3m	ELISA
Todd *et al*, 2008	Afghanistan, hospitals	4,452	1.03%	Cross sectional	4m	Commercial assay
Kumar *et al*, 2007	India, ANC	8,130	15.80%	Cross sectional	4m	ELISA, HCV RNA
Stoszek *et al*, 2006	Egypt, prenatal clinics	2,587	3.27%	Cross sectional	5m	ELISA, RT-PCR
Jaffery *et al*, 2005	Pakistan, hospital	947	0.04%	Case control	11m	ELISA, PCR
Surya *et al*, 2005	Indonesia, Hospitals	2,450	3.30%	Cross sectional	5m	Commercial assay
Simpore *et al*, 2005	Burkina Faso, ANC	547	0.73%	Cross sectional	8m	EIA
Kumar *et al, 2005*	India, ANC	1,900	0.80%	Cross sectional	9m	ELISA
Rouet *et al*, 2004	Ivory Coast, community based	501 HIV-1 negative pregnant women	4.80%	Case control	NS	EIA, HCV RNA
Khokhar *et al*, 2004	Pakistan, ANC	503	4.30%	Cross sectional	18m	ELISA, HCV RNA
Laurent *et al*, 2001	Democratic Republic of Congo, ANC	1092	1.60%	Cross sectional	NS	ELISA, LIA
Madzime *et al*, 2000	Zimbabwe, hospital	1,591	1.90%	Serological survey	NS	EIA
Njouom *et al*, 2003	Cameroon, ANC	1,494	0.50%	Cross sectional	NS	ELISA, PCR
Lewis-Ximenez *et al*, 2002	Brazil, hospital	874	1.24%	Cross sectional	2m	ELISA, Commercial assay
Lin *et al*, 1994	Taiwan, not specified	1,687	2.30%	Case control	NS	ELISA, RT-PCR
Drobeniuc *et al*, 1999	Moldova, prenatal clinic	1,098	0.31%	Cross sectional	NS	Radioimmu-noassay

m – month; y – year; ANC – antenatal clinic; ELISA – enzyme-link immunosorbent assay; RT-PCR EIA – enzyme-immunosorbent assay; LIA HCV-RNA – Hepatitis C virus RNA detection; NS – not stated; PCR – polymerase chain reaction

*Reported as the number of pregnant women who possessed anti-HCV antibodies.

†Indicates the test used to amplify HCV from patient serum.
