# Supplementary material for: Epidemiology and aetiology of maternal bacterial and viral infections in low- and middle-income countries
Source: J Glob Health. 2011 Dec;1(2):171–88. (PMC3484781)
Supplement: Supplementary Table 2 [file jogh-01-171-s002.pdf]

Supplementary table 2. Summary of data extracted from studies (n=21) reporting prevalence of maternal *Neisseria gonorrhoeae* (NG) infection

Author, Year of publication	Country, Setting of Study	Number studied	Prevalence	Study design	Duration of study	Technique used
Jalil *et al*, 2008	Brazil, prenatal services	3003	1.50%	Cross sectional	12m	Hybrid capture technique
Romoren *et al*, 2007	Botswana, ANC	703	3.00%	Cross sectional	NS	LCR
Apea-Kubi *et al*, 2004	Ghana, hospital	517	0.60%	Cross sectional	NS	RNA detection kit
Latif *et al*, 1999	Zimbabwe, ANC and primary care clinics	1189	5.80%	Cross sectional	NS	NS
Kilmarx *et al*, 1998	Thailand, ANC	1021	0.20%	Cross sectional	NS	PCR
Msuya *et al*, 2009	Tanzania, primary health clinics	2654	0.50%	Cross sectional	21m	Culture for NG
Kinoshita-moleka *et al*, 2008	Democratic Republic of Congo, maternity clinics	529	0.40%	Cross sectional	4m	PCR
Lujan *et al*, 2008	Mozambique, ANC	835	2.50%	Cross sectional	5m	PCR
Thammalangsy *et al*, 2006	Laos, hospital	500	0.80%	Cross sectional	7m	PCR
Chen *et al*, 2006	China, ANC	504	0.80%	Cross sectional	3m	PCR
Goto *et al*, 2005	Vietnam, community-based	505	0.00%	Cross sectional	NS	Culture
Amindavaa *et al*, 2005	Mongolia, prenatal clinic	2000	6.10%	Cross sectional	11m	PCR
Mayank *et al*, 2001	India, community-based	600	0.30%	Cross sectional	NS	Culture
Gray *et al*, 2001	Uganda, community-based	1394	1.70%	RCT	NS	LCR
Sullivan *et al*, 2003	Vanuatu, ANC	547	5.90%	Cross sectional	12m	PCR
Diallo *et al*, 1997	Ivory Coast, ANC	546	3.70%	Cross sectional	4m	Culture
Meda *et al*, 1997	Burkina Faso, ANC	645	1.60%	Cross sectional	NS	Culture
Mwakagile *et al*, 1996	Tanzania, ANC	777	3.60%	Cross sectional	NS	Microscopy
Joesoef *et al*, 1996	Indonesia, prenatal clinic	599	0.80%	Cross sectional	15m	Culture
Mulanga-Kabeya *et al*, 1999	Mali, community-based	549	1.00%	Cross sectional	1m	Culture
Bourgeois *et al*, 1998	Gabon, ANC	646	1.90%	Cross sectional	5m	Culture

m – month; y – year; ANC – antenatal clinic; LCR – ligase chain reaction; PCR NS – not stated; RCT – randomised control trial
