# Supplementary material for: Epidemiology and aetiology of maternal bacterial and viral infections in low- and middle-income countries
Source: J Glob Health. 2011 Dec;1(2):171–88. (PMC3484781)
Supplement: Supplementary Table 3 [file jogh-01-171-s003.pdf]

Supplementary table 3. Summary of data extracted from studies (n=39) reporting prevalence of maternal Hepatitis B virus infection

Author, Year of publication	Country, Setting of Study	Number studied	Prevalence *	Study design	Duration of study	Technique used
El-Magrahe *et al*, 2010	Libya, hospital	1500	1.50%	Cross sectional	NS	ELISA
Zhang *et al*, 2010	China, not specified	6398	6.71%	Cross sectional	23m	NS
Chatterjee *et al*, 2009	India, prenatal clinics	36379	0.82%	Cross sectional	2y 9m	ELISA, Commercial assay
De Lima & Viana, 2009	Brazil, hospital	534	1.10%	Cross sectional	9m	NS
Evelyn *et al*, 2009	Nigeria, ANC	3465	1.60%	Cross sectional/cohort study	3y	Latex agglutination assay
Sheikh, 2009	Pakistan, hospital	2592	0.34%	Cross sectional	1y	Commercial assay
Shrestha *et al,* 2009	Nepal, hospital	5602	3.21%†	Retrospective observational	6y	NS
Lin *et al*, 2008	Taiwan, hospital	10327	15.50%	Retrospective observational	5y	Microparticle EIA
Miyamoto & Bertolini, 2008	Brazil, hospital	12274	1.00%	Retrospective observational	5y	Commercial assay
Singla & Chander, 2008	India, ANC	2933	1.73%	Retrospective observational	4y	ELISA
Todd *et ali,* 2008	Afghanistan, hospital	4452	1.53%	Cross sectional	4m	Commercial assay
Elsheikh *et al*, 2007	Sudan, hospital	728	5.60%	Cross sectional	3m	ELISA
Burnett *et al*, 2007	South Africa, ANC	710	2.2%†	Retrospective case control	3m (over 3 years)	Commercial assay
Liu *et al*, 2007	Taiwan, community-based	16926	10.80%	Retrospective observational	3y	NS
Okoth *et al*, 2006	Kenya, ANC	2241	9.30%	Cross sectional observational	1y	NS
Bertolini *et al*, 2006	Brazil, prenatal service	3188	18.50%	Cross sectional	4y	ELISA
Ikeme *et al*, 2006	Nigeria, ANC	7581	0.84%	Cross sectional/screening	5y	NS
Obi *et al*, 2006	Nigeria, ANC	1499	4.60%	Prospective case-control	8m	NS
Akani *et al*, 2005	Nigeria, AND	600	4.30%	Cross sectional	NS	ELISA
Goto *et al*, 2005	Vietnam, community-based	505	10.00%	Survey	NS	ELISA
Surya *et al*, 2005	Indonesia, hospital	2450	1.90%	Cross sectional	5m	Commercial assay
Rouet *et al*, 2004	Ivory Coast, women recruited for clinical trial	501	8.00%	Case control	NS	NS
Vazquez-Martinez *et al*, 2003	Mexico, ANC	9992	1.65%	Cross sectional	4m	ELISA
Lin *et al,* 2003	Taiwan, not specified	3605	12.00%	Retrospective observational	15y	Radioimmu-noassay

Lewis-Ximenez *et al*, 2002	Brazil, hospital	874	0.50%	Cross sectional	2m	NS
Drobeniuc *et al*, 1999	Moldova, prenatal clinic	1098	9.70%	Cross sectional	NS	Radioimmu-noassay
Madzime *et al*, 1999	Zimbabwe, hospital	984	25.00%	serological survey	12m	EIA
Prakash *et al*, 1998	India, ANC	1112	9.50%	Cross sectional	NS	ELISA
Duarte *et al*, 1997	Brazil, hospital	7992	0.95%	Cross sectional	2y 10m	ELISA
dos Santos *et al*, 1995	Brazil, prenatal clinic	1024	0.60%	Cross sectional	8m	ELISA
Gill *et al*, 1995	India, ANC	2000	5.00%	Cross sectional	NS	ELISA
Oshitani *et al*, 1995	Zambia, ANC	2098	6.50%	Cross sectional	NS	NS
Woodruff *et al*, 1993	Romania, prenatal clinics	573	8.38%	Cross sectional	1m	Radioimmu-noassay
Guidozzi *et al*, 1993	South Africa, not specified	3469	1.21%	Cross sectional	NS	NS
Akhter *et al*, 1992	Bangladesh, hospital	500	3.60%	Cross sectional	3m	Commercial assay
Sebastian *et al*, 1990	Brunei, ANC	1267	3.20%	Cross sectional	NS	ELISA
Kew *et al*, 1987	South Africa, ANC	1234	2.61%	Cohort	NS	Radioimmu-noassay
Sy *et al*, 1986	Philippines, prenatal clinic	5684	7.60%	Cross sectional	17m	EIA
Anderson *et al*, 1975	Taiwan, prenatal clinic	1106	7.50%	Cross sectional	12m	Complement Fixation, Radioimmu-noassay

m – month; y – year; ANC – antenatal clinic; ELISA – enzyme-linked immunosorbent assay; EIA – enzyme immunosorbent assay; NS – not stated
*Reported as number of pregnant women who possessed Hepatits B surface Antigen (HBsAg).
†These two studies used detection of maternal HBV DNA to describe prevalence.
