# Supplementary material for: Epidemiology and aetiology of maternal bacterial and viral infections in low- and middle-income countries
Source: J Glob Health. 2011 Dec;1(2):171–88. (PMC3484781)
Supplement: Supplementary Table 4 [file jogh-01-171-s004.pdf]

Supplementary table 4. Summary of data extracted from studies (n=21) reporting prevalence of maternal Hepatitis C virus infection

Author, Year of publication	Country, Setting of Study	Number studied	Prevalence *	Study design	Duration of study	Technique used
Costa *et al*, 2009	Brazil, ANC	28,561	0.15%	Cross sectional	1 -2 yrs	EIA, HCV RNA†
De Lima & Viana, 2009	Brazil, hospital	534	1.40%	Cross sectional	9m	NS
Parthiban *et al*, 2009	India, ANC	3115	0.60%	Prospective	29m	ELISA, RT-PCR
Shebl *et al*, 2009	Egypt, community health unit	1,863	15.70%	Prospective cohort	NS	EIA, RT-PCR
Sheikh, 2009	Pakistan, hospital	2,592	0.69%	Cross sectional	1y	Commercial assay
Ndong-Atome *et al*, 2008	Gabon, ANC	947	2.10%	Cross sectional	3m	ELISA
Todd *et al*, 2008	Afghanistan, hospitals	4,452	1.03%	Cross sectional	4m	Commercial assay
Kumar *et al*, 2007	India, ANC	8,130	15.80%	Cross sectional	4m	ELISA, HCV RNA
Stoszek *et al*, 2006	Egypt, prenatal clinics	2,587	3.27%	Cross sectional	5m	ELISA, RT-PCR
Jaffery *et al*, 2005	Pakistan, hospital	947	0.04%	Case control	11m	ELISA, PCR
Surya *et al*, 2005	Indonesia, Hospitals	2,450	3.30%	Cross sectional	5m	Commercial assay
Simpore *et al*, 2005	Burkina Faso, ANC	547	0.73%	Cross sectional	8m	EIA
Kumar *et al, 2005*	India, ANC	1,900	0.80%	Cross sectional	9m	ELISA
Rouet *et al*, 2004	Ivory Coast, community based	501 HIV-1 negative pregnan t women	4.80%	Case control	NS	EIA, HCV RNA
Khokhar *et al*, 2004	Pakistan, ANC	503	4.30%	Cross sectional	18m	ELISA, HCV RNA
Laurent *et al*, 2001	Democratic Republic of Congo, ANC	1092	1.60%	Cross sectional	NS	ELISA, LIA
Madzime *et al*, 2000	Zimbabwe, hospital	1,591	1.90%	Serological survey	NS	EIA
Njouom *et al*, 2003	Cameroon, ANC	1,494	0.50%	Cross sectional	NS	ELISA, PCR
Lewis-Ximenez *et al*, 2002	Brazil, hospital	874	1.24%	Cross sectional	2m	ELISA, Commercial assay
Lin *et al*, 1994	Taiwan, not specified	1,687	2.30%	Case control	NS	ELISA, RT-PCR
Drobeniuc *et al*, 1999	Moldova, prenatal clinic	1,098	0.31%	Cross sectional	NS	Radioimmu-noassay

m – month; y – year; ANC – antenatal clinic; ELISA – enzyme-link immunosorbent assay; RT-PCR EIA – enzyme-immunosorbent assay; LIA HCV-RNA – Hepatitis C virus RNA detection; NS – not stated; PCR – polymerase chain reaction

*Reported as the number of pregnant women who possessed anti-HCV antibodies.

†Indicates the test used to amplify HCV from patient serum.
